# Mechanical Nano-Patterning: Toward Highly-Aligned Ge Self-Assembly on Low Lattice Mismatched GaAs Substrate

**DOI:** 10.1038/s41598-019-50633-y

**Published:** 2019-10-02

**Authors:** Ghada Dushaq, Mahmoud Rasras

**Affiliations:** Department of Electrical and Computer Engineering, New York University Abu Dhabi, P.O. Box 129188, Abu Dhabi, UAE

**Keywords:** Materials for optics, Lasers, LEDs and light sources, Semiconductor lasers, Materials science, Nanoscale materials, Quantum dots, Nanoscience and technology, Design, synthesis and processing, Raman spectroscopy, Applied physics

## Abstract

Low-dimensional semiconductor structurers formed on a substrate surface at pre-defined locations and with nano-precision placement is of vital interest. The potential of tailoring their electrical and optical properties will revolutionize the next generation of optoelectronic devices. Traditionally, highly aligned self-assembly of semiconductors relies on Stranski- Krastanov growth mode. In this work, we demonstrate a pathway towards ordered configuration of Ge islands on low lattice mismatch GaAs (110) substrate patterned using depth-controlled nanoindentation. Diamond probe tips with different geometries are used to nano-mechanically stamp the surface of GaAs (110). This creates nanoscale volumes of dislocation-mediated deformation which acts to bias nucleation. Results show that nanostamped GaAs exhibits selective-nucleation of Ge at the indent sites. Ge islands formed on a surface patterned using cube corner tip have height of ~10 nm and lateral size of ~225 nm. Larger islands are formed by using Vickers and Berkovich diamond tips (~400 nm). The strain state of the patterned structures is characterized by micro-Raman spectroscopy. A strain value up to 2% for all tip geometries has been obtained. Additionally, strong room temperature photoluminescence (PL) emission is observed around 1.9 µm (650 meV). The observed strain-induced enhancement in the light-emission efficiency is attributed to direct conduction to heavy-hole (cΓ-HH) and conduction to light-hole (cΓ-LH) transitions. The inherent simplicity of the proposed method offers an attractive technique to manufacture semiconductor quantum dot structures for future electronic and photonic applications.

## Introduction

Nanoscale templating of semiconductor surface has received tremendous research attention in the recent years^1–3^. This technique utilizes surface modifications such as pits, strain field modulation and oxide window as the primary mechanisms for site-selective nucleation of low-dimensional semiconductor nanostructures. This approach is of extreme importance for the realization of novel electronic and photonic device technologies, including high efficiency quantum dot lasers^4,5^, multi-spectrum quantum optoelectronics^6^ and high density memory storage^7^. A variety of methods have been developed to nano-pattern semiconductor surface. For instance, reactive ion etching (dry etching) or chemical etching on a semiconductor surfaces, which is previously treated with direct or in-direct writing techniques, have been demonstrated. This includes photolithography, e-beam lithography, dip-pen nanolithography, and soft lithography. However, those techniques still suffer from a number of drawbacks, in particular, the limitation of their spatial resolution for dimensions smaller than 10–20 nm^3^. Additionally, an effective and low cost fabrication process for achieving precise placement of uniform nanostructures has not been realized yet^8^.

Instrumented nano-indentation is a widely utilized technique and a well-controlled method for measuring the mechanical properties of bulk materials and thin films^8–11^. This technique allows for understanding the mechanical response of materials by applying a highly localized mechanical deformation. Interestingly, it has been recently refined in order to manipulate the sample surface on the nanoscale level. For instance, controlled growth of self-assembled nanostructures of InAs on GaAs and Ge on Si have been successfully demonstrated^12,13^. This maskless technique allows patterning of large areas via localized dislocations without the need for chemical/dry processing. Thus, surface nano-stamping can be performed as a complete physical method.

**Figure 1:**
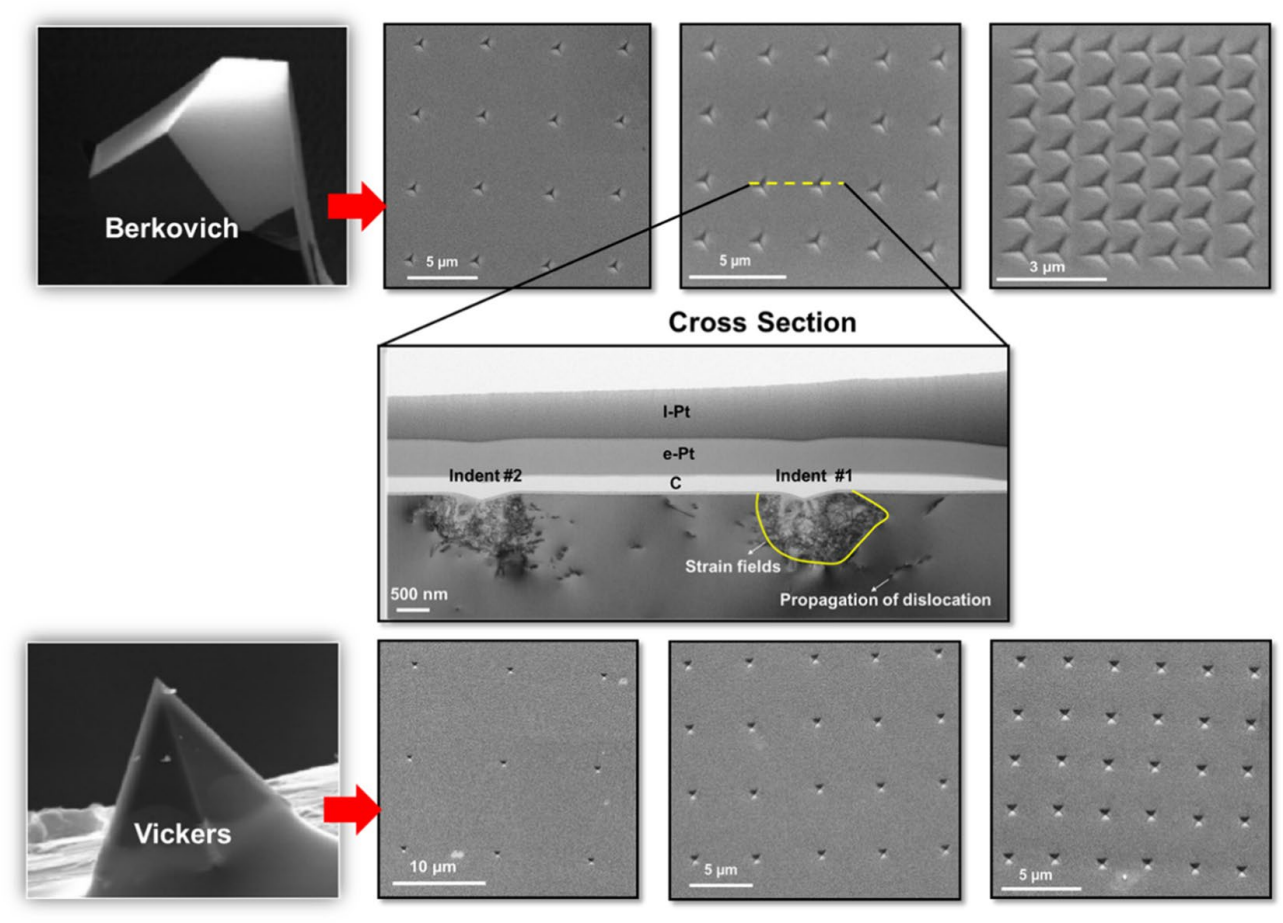
SEM top view images of GaAs (110) substrate patterned using depth controlled nanoindentation using Berkovich and Vickers diamond indenter tips. The images show indentation performed with maximum penetration depth of 150 nm and different indents spacing in *x*, *y*. SEM images of the diamond tips^52^. A TEM cross-section image of the indents shown in dashed yellow line is described, the strain field and the dislocation propagation beneath the indents are clearly observed.

Furthermore, it is well known that nano-indentation creates highly-localized stresses under diamond indenters which results in pressure-induced phase transformations. This clearly observed in the case of silicon^14,15^. Additionally, deformation controlled by the formation and propagation of defects, such as slip and twinning, has been observed in GaAs and Ge^16,17^. Nano-stamping technique regulates the surface strain with adequate control over feature dimensions by using different diamond tip geometries. Thus, it allows precise placement of nanostructures on the growth surface. Figure 1 shows Scanning Electron Microscope (SEM) images and a Transmission Electron Microscope (TEM) cross section of nano-stamped structure performed on GaAs (110) substrate using Berkovich (three-sided pyramid) and Vickers (four sided pyramid) diamond tips. As can be seen in this figure, by controlling the applied load and the choice of appropriate tip geometry, the plastic deformation size, dislocation density, and hence subsequent strain field can be regulated.

Traditionally, self-assembly of Ge dots on Si or InAs dots on GaAs mainly relies on Stranski- Krastanov growth mode^18,19^. The finite lattice mismatch between the two materials is the origin of excess elastic strain energy. This leads to the formation of nanometer-scaled islands which is usually referred to as a quantum dot^20^. In particular, tensile stress applied to Ge has the potential to improve light emission by modifying the electronic band structure^21,22^. By introducing high tensile strain to germanium crystal, a crossover of the Γ minima and L minima of the conduction occurs. Thus, it transforms this indirect band gap material into a direct gap semiconductor. However, the maximum Ge strain that can be introduced in epitaxial growth is quite small (0.25%) and not sufficient for the development of high quality Ge lasers (the ultimate goal of group-IV photonics)^23^.

In this paper, we present an effective pathway towards ordered configuration of Ge islands on low lattice mismatch GaAs (110) substrate patterned by depth-controlled nanoindentation. The mismatch of the lattice parameters of GaAs and Ge is only 0.07% and the nucleation of Ge layer on GaAs typically occurs through a 2D mechanism. Thus, utilizing highly localized stress and strain induced by nanoindentation offers a novel technique to nucleate low lattice mismatch materials. Additionally, we show that careful consideration of the tip geometry allows for greater control of the applied surface stress/strain and surface feature parameters.

The first part of the paper focuses on the nano-mechanical stamping process as a technique to regulate surface strain and bias Ge nucleation on low lattice mismatch GaAs substrate. By investigating the evolution of the Ge islands on GaAs, we gain experimental insight into the initial stage of the ordering process. Results are explained in terms of the spatial distribution of the induced pressure for different indenter geometries. Also in terms of its correlation to the Raman peak shifts in Ge islands. Each tip geometry creates a different applied average stress and strain gradient on the contacting surface, thus it allows tailoring the strain field on the substrate surface.

In the second part of the paper, room temperature photo-luminescent of Ge islands has been tested. The observed strain-induced light emission enhancement agrees well with the theoretical prediction, revealing that the direct band gap of Ge can be tuned to 0.658 eV (1885 nm). These results describe an attractive, inherently simple and elegant method towards realizing strained Ge laser with low threshold current. Additionally, it opens possibilities for new types of high-speed optoelectronics devices based on a complete physical strain engineering process.

## Results and Discussion

### Mechanical nano-stamping

**Figure 2:**
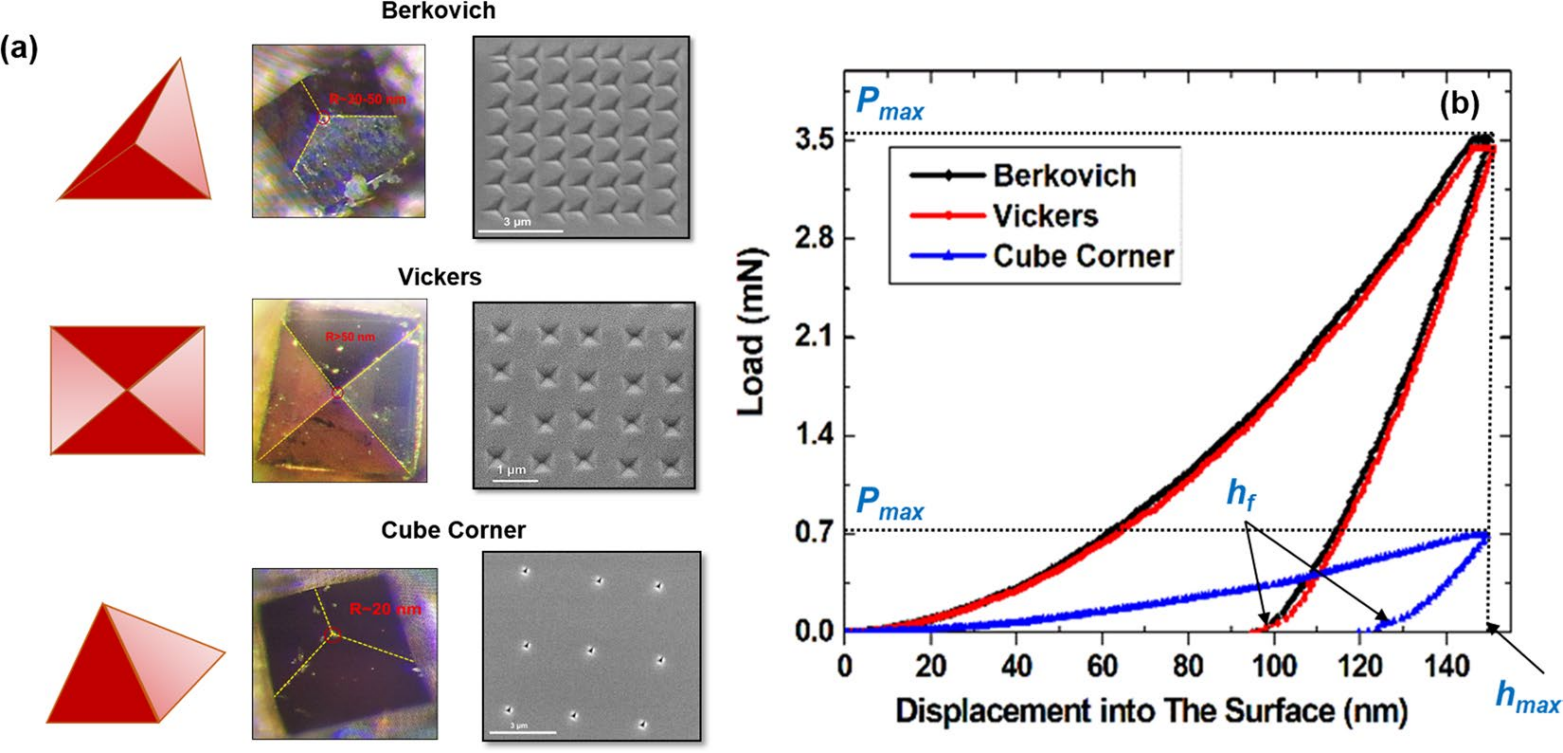
Mechanical Nano stamping (**a**) SEM top view images of GaAs (110) substrate patterned using depth controlled nanoindentation using Berkovich, Vickers and cube corner diamond indenter tips. The images show indentation performed with maximum penetration depth of 150 nm and different indents spacing in *x*, *y*. Optical microscope images and schematic representation of the diamond tips^52^. (**b**) Load-Displacement curves (*P*-*h*) probed using different tip geometries on GaAs (110) substrate.

Figure 2a shows SEM images of the residual impression of the indents array on GaAs (110) surface. The indentation is performed at 150 nm depth. Using simple calculations, the projection area can be determined based on the indents shape and geometries. The 150 nm is the maximum tip penetration depth defined by the user. The area of contact at full load is calculated from the known angle or radius of the tip as measured by the HRTEM^9^. The projected area functions A(*h*_*c*_) for perfect Berkovich, Vickers and cube corner tips are: 3(3)^1/2^*h*_*c*_^2^tan^2^*θ*, 4 *h*_*c*_^2^tan^2^*θ*, and 3(3)^1/2^*h*_*c*_^2^tan^*2*^*θ*, respectively^24^. *h*_*c*_ is the contact depth, this figure is different from the maximum penetration depth *h*_*max*_ due to the elastic recovery of the GaAs surface after indentation. To find the projection area, we extracted the contact stiffness *S* from the slope of load-displacement (*P*-*h*) curves, as shown in Fig. 2b. The final depth *h*_*f*_, maximum load (*P*_max_) and the maximum depth *h*_*max*_ (150 nm) were also extracted and inserted into this equation (*h*_*c*_ = *h*_*max*_ − 0.75 × (*P*_*max*_/*S*))^9,24^. Table 1 shows the approximate values of the calculated indents projection area, contact depth and the substrate mechanical parameters.

**Table 1:** The mechanical parameters and projected area of GaAs (110) indented using different tip geometries calculated from *P*-*h* curves based on Oliver & Pharr method^9,24^.

Tip Type	Max. Force (mN)	Hardness (GPa)	Young’s Modulus (GPa)	h_c_ (nm)	Aproj (nm^2^)	Pmax (GPa)	Tendency to Pop-ins and outs
Berkovich	3.44 ± 0.07	9.4 ± 0.2	140 ± 4	118.54	344127	10	Yes
Vickers	3.33 ± 0.06	9.4 ± 0.3	136 ± 4	116.53	332746	10	Yes
Cube Corner	0.69 ± 0.02	1.47 ± 0.04	46 ± 2	139.1	50307	13.7	Yes

The average contact pressure values at the early stages of indentation are high and then rapidly decreases during the loading with the penetration displacement. Following that it quickly reaches a constant value. Using the calculated area values, the induced contact pressure for different tip geometries are determined. According to Table 1, Berkovich and Vickers showed almost the same parameters. However, cube corner tips exhibited the lowest maximum applied force to produce a plastic deformation on the surface. Furthermore, the elastic response of the GaAs (110) is very sensitive to the shape of the indenter as depicted in Table 1. This can be explained based on the tip interaction volume characteristics. For instance, for the same penetration depth, shallow Berkovich indentation imprints are almost two times bigger in the edge length compared to cube-corner ones. Thus, Berkovich indenter probes ~8-times bigger volume^25^.

It is important to point that for pyramid-shaped tips the pressure distribution is not uniform since the contact effective shape changes at different points inside the indents^26–28^. More details will be discussed in the Raman shift section.

The loading curves of 49 indents were analyzed. The pop-in and pop-out events in *P*-*h* curves are usually indicative of the type of mechanical deformation process in covalent semiconductors. A pop-in event occurs as an indication of slip, twinning and shear activities^16,29^. However, a pop-out event is linked to the formation and nucleation of other GaAs crystalline phases such as orthorhombic Cmcm^30,31^. The primary reason for those events is a sudden change in a discrete material volume from high to low density phases. It has been predicted that GaAs transforms to orthorhombic Cmcm phase at about 12.5 GPa. A complete transformation was achieved in pressure cell experiments at 17.5 GPa^32^. These figures are significantly higher than the room-temperature GaAs hardness values (see Table 1). As can be seen in Fig. 2b, some of the analyzed curves were featureless where no pop-in events have been observed. However, others exhibited pop ins in the loading part. On the other hand, no pop-out events were observed in all the unloading curves. This is in agreement with the TEM cross section shown in Fig. 1. It implies that the indentation behavior of GaAs is most likely controlled by dislocation gliding similar to that of metals^31,33,34^.

### AFM study of Ge islands formed on the GaAs (110) surface

**Figure 3:**
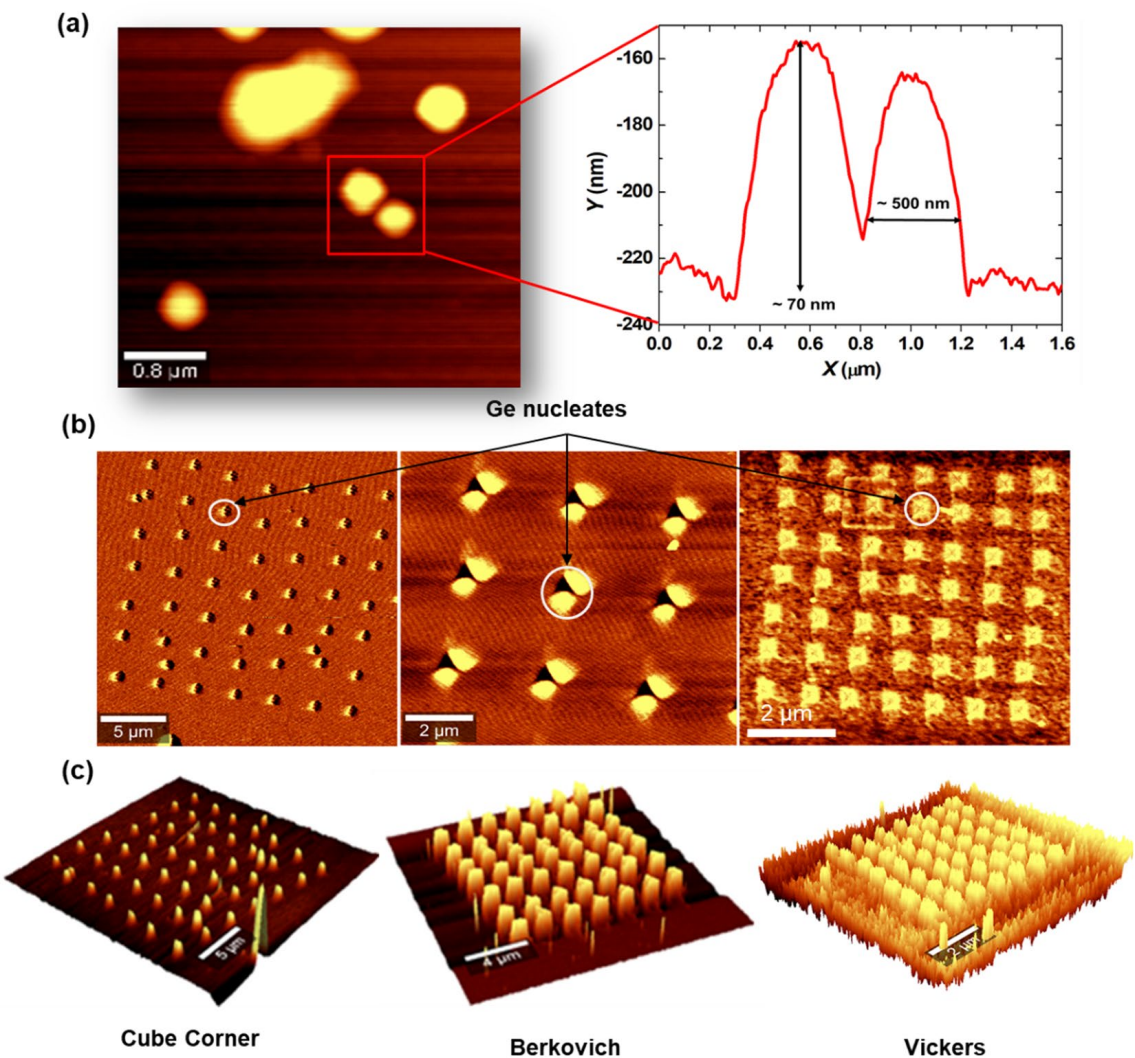
AFM scan images of Ge islands on GaAs (110) (**a**) un-patterned surface (**b**) patterned surface using different tips geometry (**c**) 3D representation restructured from the height imaging using AFM of indented surface using different tips geometry.

Plastic deformation creates a collection of dislocations inside the indentation site (pits). Such dislocations and surface deformation provide a preferential sites for the Ge growth. AFM scan images of Ge nucleation have been carried out on un-patterned and patterned GaAs surfaces. As shown in Fig. 3a, the un-patterned surface depicts micron scale non-uniform distribution of random shaped islands. This occurs due to the fact that the lattice parameter mismatch between GaAs and Ge is only 0.07% and the nucleation typically occurs through a 2D mechanism^35^. Thus, the tendency of large Ge island formation occurred randomly. However, patterned surface exhibited selective-nucleation process as depicted in Fig. 3b. Here Ge primarily nucleates on the indents edges (depending on tip shape) and part of it diffuses to the indent side wall which resulted in a Ge islands formation at those locations. Figure 3c shows 3D topographical images of Ge on nano-indented GaAs surface.

**Figure 4:**
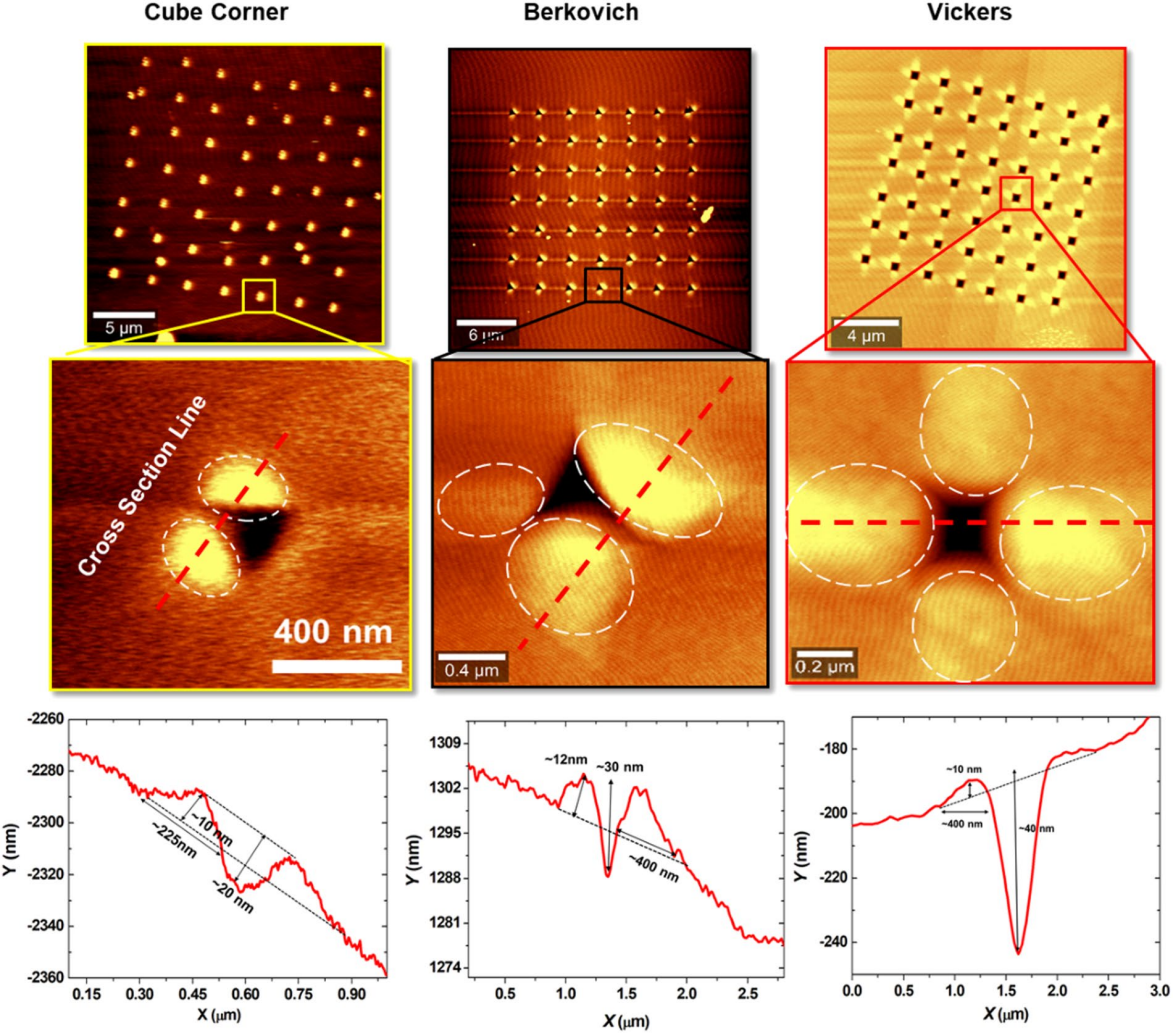
AFM image scan (low and high magnification) of Ge nucleates on GaAs surface patterned using different tip geometry, high magnification images show how Ge nucleates follow the strain fields of the indent, additionally line cross section (red dashed line) of each tip is represented in the *X*,*Y* curves.

The effect of mechanical stamping and strain on the Ge feature size, morphology, and spatial distribution is depicted in Fig. 4. Ge islands formed on a surface patterned with cube corner tip exhibited height and lateral size of ~10 nm and ~225 nm, respectively. Lager islands were formed using Vickers and Berkovich tips as depicted in the AFM cross section line scans in Fig. 4. The features were imaged by *ex-situ* AFM, the measured depths represent residual impressions that comprise plastic recovery effects. Consequently, an indentation depth <150 nm was observed with values dependent on the tip geometry. The resulting structure of indentations signified that cube corner tip can provide a more coherent strain field and hence higher precision dot formation. By applying different loading conditions, the plastic deformation size, dislocation density, and hence subsequent strain field can be regulated. Perhaps the most notable parameters among these is the depth of the nucleation site, thus shallow indentation can result in a highly controlled Ge dots with smaller lateral sizes.

### Micro-Raman spectroscopy

**Figure 5:**
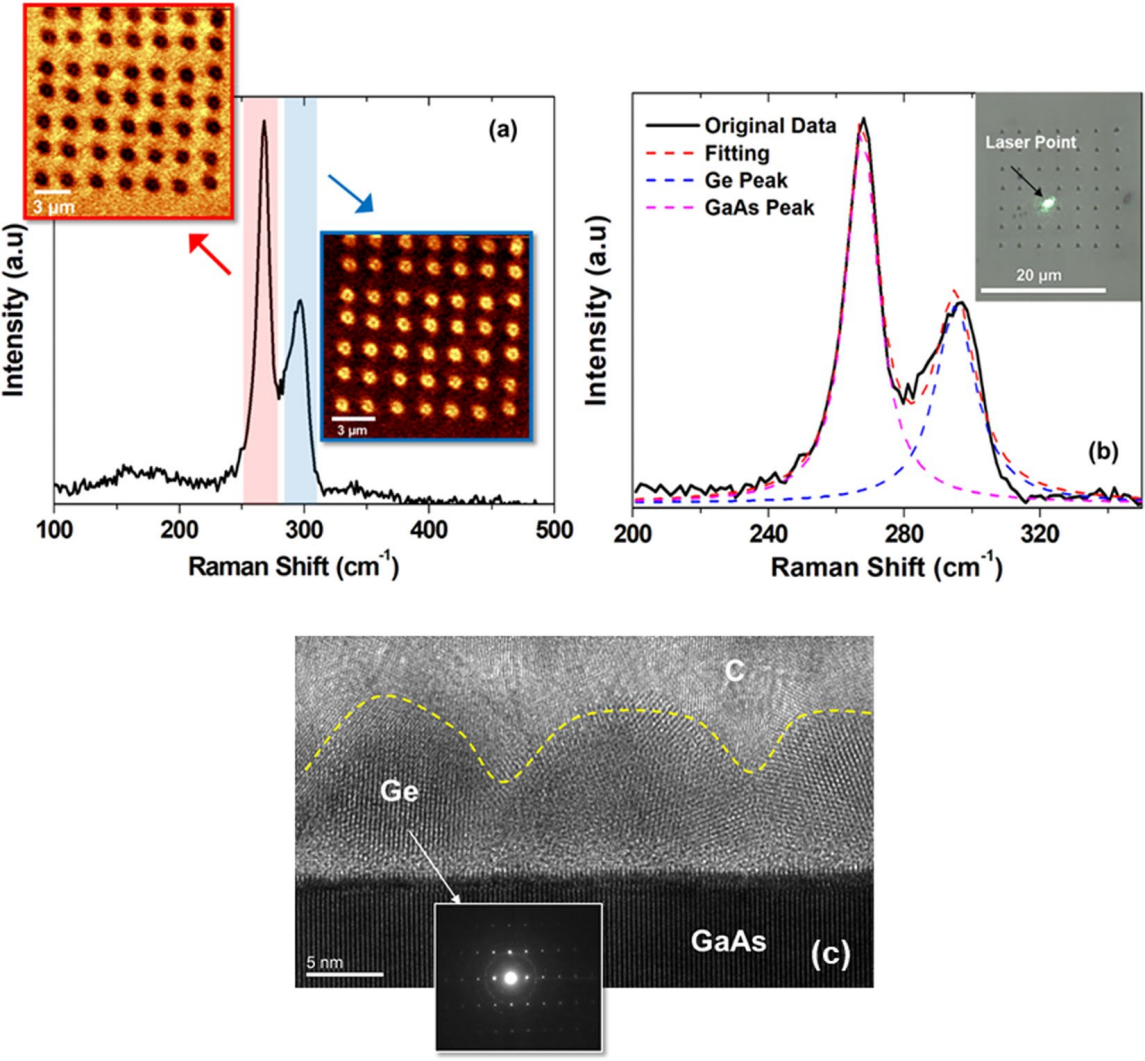
Raman spectra of Ge nucleates on GaAs (110) surface patterned using Berkovich tip (**a**) Raman Mapping of 20 µm × 20 µm indents array with red and blue filters define where the mapping was scanned (**b**) Lorentz- Gaussians fitting with inset showing laser spot aligned to the indents location using 100x optical microscope objective (**c**) XTEM image showing Ge island on (110) GaAs along with SADP of the area indicated by the white arrow.

Another key factor essential to Ge islands growth and has large influence on tailoring their optoelectronic properties is the residual strain. Using micro-Raman spectroscopy, the strain induced on the Ge islands has been characterized. The strain is directly determined from the frequency shift in the Raman-allowed singlet component of the optical phonons according to the following equation^36–39^:

1 $${\omega }_{0}={\omega }_{{\rm{s}}}+{{\rm{b}}}_{{\rm{s}}}\Delta \varepsilon .$$

here ω_0_ and ω_s_ are the unstrained and singlet component angular frequencies, *ε* is the elastic deformation (strain) leading to the shift of the optical phonon frequency with respect to the 0 state and b_s_ is the phonon strain-shift coefficient. Such strain-induced shifts are well known from Raman measurements^36–39^. It indicates that the optical phonon frequencies linearly increase/decrease with compressive/tensile strain. We can approximate the local strain in the area under the laser illumination by using the distinct relation between longitudinal optical (LO) shifts and strain in Ge nucleates. The amount of peak shift (Δω) is calculated between the experimentally measured relative to the ones obtained from the unstrained LO frequency of Ge (300.8 cm^−1^)^40^. Figure 5a shows Raman spectrum of Ge nucleates on a GaAs (110) surface patterned using Berkovich tip. Additionally, Raman mapping of 20 µm × 20 µm indents array is depicted using image scan filters. The Raman laser spot is aligned to the indents location using 100x optical microscope objective as shown in the inset of Fig. 5b. Two peaks were observed. The one at ~267.6 cm^−1^ is the only transverse optical (TO) mode which is typically observed from backscattering in 110 zincblende GaAs surfaces^41^. The other one, at ~295 cm^−1^, corresponds to the shifted Ge–Ge mode peak generated due to biaxial stresses^20,21,40^. Using Eq. 1, Table 2 shows the calculated Raman shift (Δω) and their corresponding strain values for other tip geometries.

**Table 2:** Raman shift obtained from the subtraction of LO phonon frequency of un-strained Ge and the fitting of experimental peak position in Raman spectra, additionally the strain percentage calculated based on Eq. 1 and b values (408 cm^−1^) in^37,39^.

Tip Type	Δω = ω_0_ − ω (cm^−1^)	ε (%)
Un-strained Ge	0	0
Berkovich	−8 ± 1	1.98 ± 0.24
Vickers	−8 ± 1	1.98 ± 0.24
Cube Corner	−13 ± 1	3.2 ± 0.4

In order to study the microstructure of the Ge island in the vicinity of indented zone, as well as their shape and crystallinity, a bright field cross sectional transmission electron microscopy (XTEM) images are carried out. Figure 5c shows the extent of crystal structure of Ge island on (110) GaAs. No misfit dislocation or crystal defects are observed at the hetero-interface of the two materials. This is consistent with the fact that the lattice parameter mismatch between GaAs and Ge is negligible and the nucleation typically occurs through a 2D mechanism. Additionally, selected area diffraction patterns (SADP) taken from the area indicated by the white arrow (see inset of Fig. 5c). The SADP mainly contain strong diffraction points which are linked to crystalline (c-Ge) phase, in addition to small grains of polycrystalline Ge. Crystalline Ge is expected in our method since *in-situ* annealing at 600 °C has been performed as explained in the germanium deposition & nucleation section of the method.

As can be seen in Table 2, Ge assembly grown on a surface patterned by cube corner exhibites the highest strain value among all indenters. This is consistent with previous reports on cube corner deformation analysis^12,25^. In the context of Raman analysis, we can deduce that induced strain introduced on Ge assembly follow the same stress-strain maps presented in the pre-nanopatterning. Thus, this physical technique offers a very promising strain engineering process of Ge assembly.

### Photoluminescence measurements (PL)

**Figure 6:**
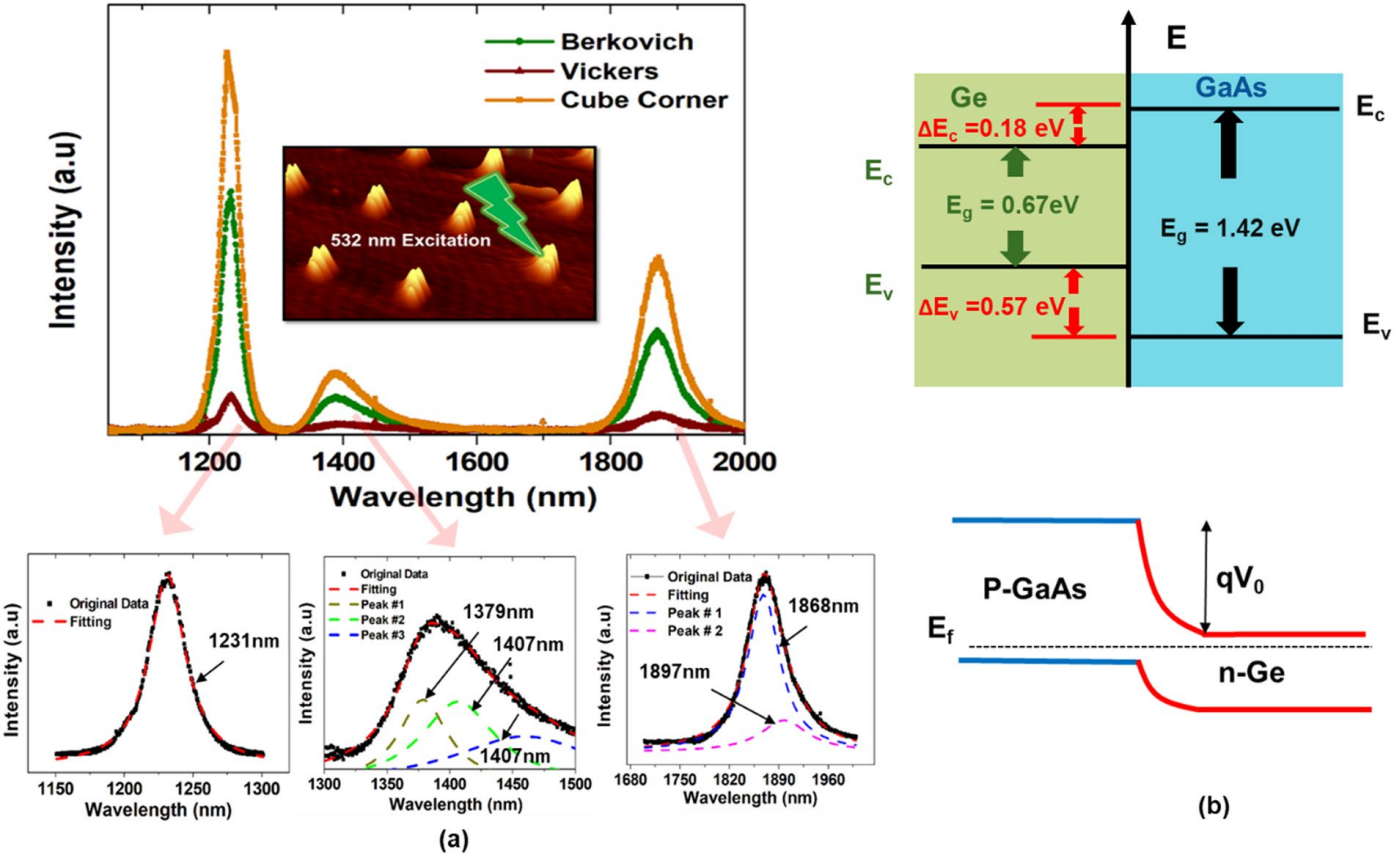
PL spectra of Ge on (110) GaAs (**a**) PL emission of Ge assembly grown on nano-indented (110) GaAs surface using different tips geometry, multi-peak fitting using Lorentz- Gaussians models was carried out for each peak (**b**) band diagram alignment at the hetero-junction Ge/GaAs. The conduction band offset ΔE_c_ and valance band offset ΔE_v_ were obtained from Hudait *et al*.^53^.

The PL measurements are carried out to study the radiative recombination mechanisms of excess carriers in the grown Ge assembly. Furthermore, the nature and the occurrence of radiative transitions, intermixing, and dopants have been also studied. Figure 6a shows the PL spectra of the Ge islands using different tip geometries. All spectra show similar peak positions and shapes, while intensity variation is observed. The spectra consists of three main peaks, one at wavelength of ~1.23 µm (1.01 eV), and the other two peaks were identified using multi-peak Lorentz- Gaussians fitting. As can be seen in Fig. 6a, the peak around 1.4 µm was fitted using three peaks with maxima at (i) 1.38 µm (0.899 eV) (ii) 1.41 µm (0.879 eV) and (iii) 1.46 µm (0.849 eV). While the lowest energy one, around 1.9 µm (650 meV), was fitted using two peaks with energy difference of ~7 meV.

We attributed the peak at 1.01 eV to the deep trapping states arise from GaAs-based complexes. It is formed within the Ge at the Ge–GaAs hetero-interface. This attributed to a strong inter-diffusion of Ga and As atoms into the Ge. Previously, the peaks at 1.04 eV–1.17 eV were identified to be due to a GeGa–GeAs and GeGa-VGa complexes^42^.

A broad luminescence peak found at about 1.4 µm (1.38 µm, 1.41 µm, and 1.46 µm) can be attributed to the direct band gap transitions between the C-valley electrons in the GaAs matrix and the valence band heavy-holes in the Ge assembly. A peak at about 1.3 µm has been observed previously in Ge quantum wells grown on GaAs^43^. The lateral sizes of our Ge assembly are more than 200 nm and they provide features similar to 2D films. However, the red shift observed in our luminescence peak can be linked to defects appearing due to very heavy intermixing between Ge and GaAs during the growth. It is also attributed to the built in electric field created by the heterojunction between the two materials. Accordingly, the carrier separation (space charge region at the hetero-interface) can contribute to the observed low peak intensity. Figure 6b describes the band diagram of GaAs/Ge hetero-structure where the Fermi level is adjusted between the two material resulting in E_0_ = qV_0_. Additionally, other effects such as valence band mixing, depolarization, exciton-like shifts, strain distribution in and around the island and their dependence on the growth conditions, all can contribute to the observed red shift.

Interestingly, strong room temperature PL emission is observed around 1.9 µm as depicted in Fig. 6a. Ge is an indirect bandgap material, so an electron transits from the conduction band to the valence band mainly by phonon-assisted recombination with non-radiative process. This results in slow and inefficient direct optical recombination. However, tensile strain has been theoretically predicted to lower the conduction band edge at the direct (Γ) point relative to the L valleys, while the overall bandgap energy correspondingly decreases^23,44,45^. This behavior is consistent with the measured PL peak at 1.9 µm (0.65 eV). This results in more photoexcited electrons to thermalize near the Γ minimum, where they can efficiently recombine via inter-band light emission. In all strained samples the PL emission wavelength was similar when using different tip geometries, except intensity variation. This might be related to the laser spot alignment with the Ge nucleates in addition to the size and shape discrepancies. In Table 2 we reported a strain value ~>2% for all tips geometry. Typically, at this high strain, indirect transitions are suppressed because of the decreasing number of electrons in the L valleys. Correspondingly, we attributed the PL emission at 1.9 µm to direct conduction to heavy-hole (cΓ-HH) and conduction to light-hole (cΓ-LH) transitions. With these assignments, the agreement between the experimental peak emission and the theoretical bandgap energies is quite good.

Our Ge assembly exhibits high photo-luminescent at room temperature, indicating enhanced light-emission efficiency. Traditionally, the use of strain engineering to manage group IV light source has relied on the epitaxial growth of overlayers with sufficiently small thicknesses (to avoid dislocation formation) on lattice mismatched substrates. Furthermore, some recent work has focused on strain technologies for Ge such as nanomembranes (NMs)^23^, Ge microstructures^46,47^ and depositing stressor layers^48^. Based on those techniques, a state-of-the-art strain values of 4.9% and 1.9% under uniaxial and biaxial stress have been achieved, respectively. Therefore, we believe that using nano-indentation as a complete physical strain engineering technique is very attractive for applications in active media of the near-IR light emitters.

It’s noteworthy that at 532 nm laser source excitation, the absorption coefficient of Ge is equal to 5.8 × 10^5^ cm^−1^^49^. The ratio between transmitted light intensity I_t_ and incoming light intensity I_0_ through a film of thickness d can be expressed as^50^:

2 $${{I}}_{{t}}={{I}}_{{0}}{\exp }(-\alpha {d})$$

where α is the absorption coefficient. From this equation we can calculate that about 56% of all incoming 532 nm photons are absorbed in ~10 nm thick Ge islands. Therefore, we expect stronger PL enhancement using a cap layer on the top surface. This thin layer reduces surface recombination and can result in strong light enhancement efficiency at room temperature^51^.

## Conclusion

In summary, nanomechanical stamping using diamond tips with different geometries was utilized to regulate the surface strain of GaAs (110) substrate. The strained sites act as a host for Ge nucleation, hence allowing selective growth of directed Ge assembly on law lattice mismatched substrate. The results show that, by the careful selection of probe tip geometry and indentation depth, nanostamped feature geometry, size, and position can be controlled with nanometer resolution. Among the three diamond tips used in this study, cube corner can provide a more coherent strain field and hence higher precision dot formation. The assembly formed on a surface patterned using this tip has a height of ~10 nm and lateral size of ~225 nm. Larger islands are formed using Vickers and Berkovich diamond tips. The strain state of patterns produced with different tips were characterized by micro-Raman spectroscopy. The Ge-Ge phonon oscillation peak exhibited a red shift in all tested samples due to the tensile strain in the underlying template pattern. Due to the high tensile strain values in Ge assemblies, the conduction band edge at the direct (Γ) point was reduced relative to the L-valleys, while the overall bandgap energy correspondingly decreases. This is in agreement with the observed strong room temperature PL emission at 1.9 µm (650 meV). In summary, this complete physical technique is very attractive for forming active media of near-IR light emitters. Additionally it opens up the possibility of directly patterning quantum structures of different sizes on the same surface for applications in multicolor surface emitters and displays.

## Method

### Sample preparation

A (110) P-type GaAs substrate (Zn-doped) are cleaned using acetone, isopropanol and DI water to achieve high-quality GaAs surface which is free from contamination and clear to start the nano-indentation.

### Indentation patterning

The nano-mechanical stamping on GaAs surface was carried out using Agilent Technologies Nano Indenter G200. The indenter stage is piezo-electrically driven Z-flexure. It is powered by electromagnetic actuation-based force transducers to ensure precise measurements. The instrument’s unique design avoids lateral displacement artifacts, while the software compensates fully for any drift in force.

The samples were patterned using diamond tips of different shapes: Berkovich, Vickers and cube corner-type indenters. In the Berkovich case, the pyramid is constructed to meet at a single point with a tip nominal radius of 30 nm and face angle of 65.3°. The four-sided Vickers pyramid has a tip radius >50 nm and a face angle of 68.12°. The cube corner has a tip radius of ~20 nm and a face angle of 35.15° which was measured using an AFM and high-resolution TEM. The analysis of the patterned sample surface, its size, and mechanical properties are significantly affected by the tip rounding and the indenter geometries.

Using a single cycle automated indentation process, we generated two dimensional ordered square arrays of 49 (7 × 7) indents. A diamond tip with surface approach distance of 1 μm and fixed velocity of 10 nm/s were used. The load function was defined to adjust its force till it reaches the maximum defined depth of 150 nm, then it retracts at a user defined speed. A maximum indentation depth of 150 nm was used throughout the paper to form inverted pyramidal impressions. The indents lateral size was varied from 200 nm to 900 nm, depending on the indenter geometry, and carried out at an ambient temperature of 27 °C. A loading and unloading rates of 0.27–0.57 mN.s^−1^ with holding time of 10 s have been used. Additionally, the tests were performed where the load was partially released after loading with unloading percentage of 90% of the maximum.

### Germanium deposition & nucleation

**Figure 7:**
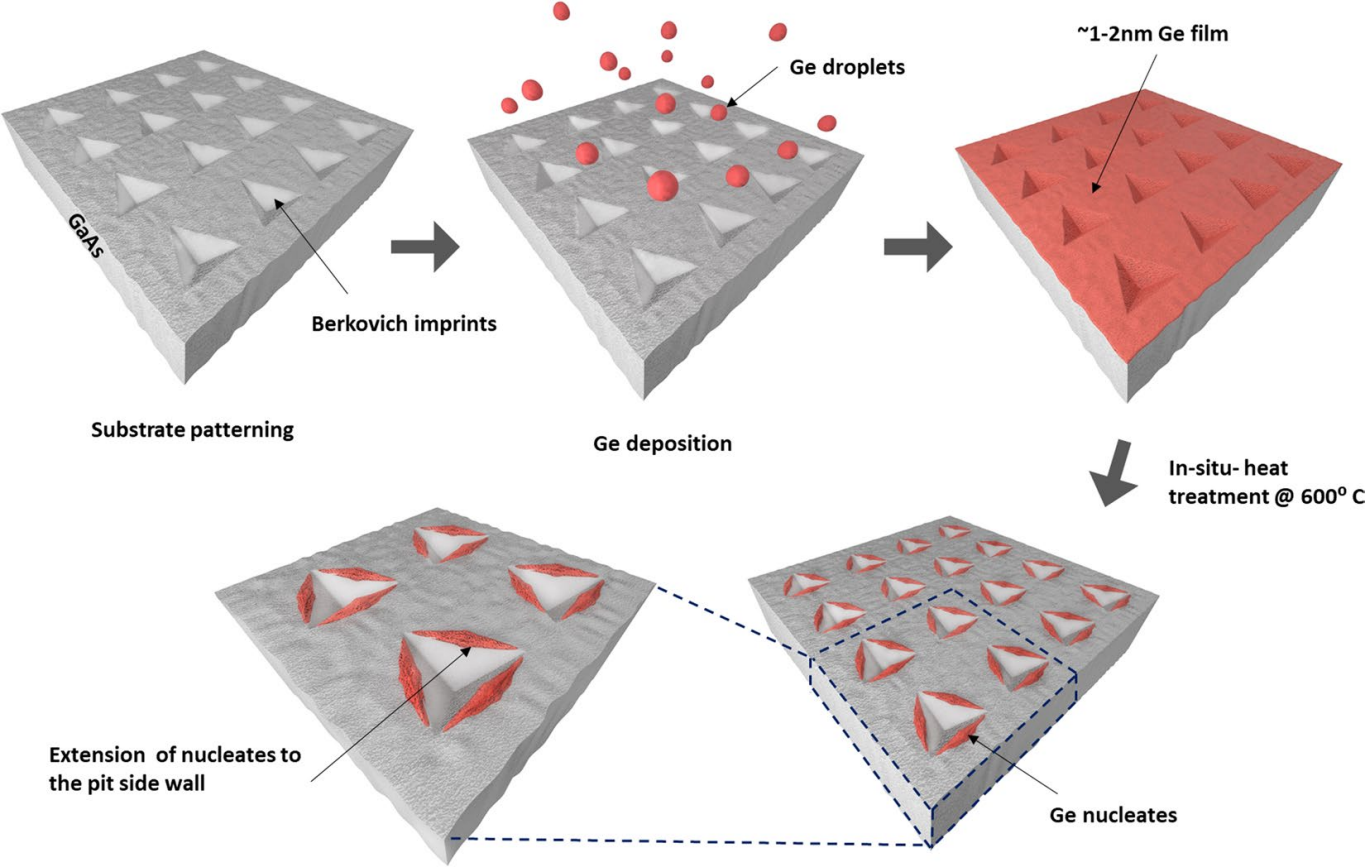
Preparation process of Ge assembly on (110) GaAs substrate.

The Ge layer was deposited on the GaAs surface using the Kurt Lesker Proline PVD 75 e-beam evaporator. The deposition was performed at room temperature using high purity 99.99% Ge pellets with chamber pressure of 1 × 10^−6^ torr and deposition rate of 1 Å/sec. After a deposition of ~2 nm Ge, *in-situ* heating was carried out by raising the samples stage temperature to 600 °C. Heat was applied over the sample for one hour before the cooling down process started. Figure 7 shows a schematic of the indentation, the Ge deposition process, and the nucleation mechanism performed on the GaAs surface. The Ge islands nucleate both inside and around the indent edges (following maximum tensile strain map). In particular, they gather at the corners and on the slopes of the pit sidewalls.

### Material characterization

#### TEM and FIB Imagining

In order to observe the deformation process beneath the indenter tip, a bright field cross sectional transmission electron microscopy (XTEM) images of selected indents are carried out using FEI Tecnai TF-20 FEG/TEM operated at 200 kV in bright-field (BF). A TEM-ready sample was prepared using an *in-situ* FIB lift out technique using FEI Dual Beam FIB/SEM. The sample was capped with sputtered carbon (C) and e-Pt/I-Pt prior to milling to protect the surface of the area of interest.

#### Micro-Raman spectroscopy & AFM combined system

The indentation impression was examined using combined WItec alpha 300 Raman micro-spectrometry and AFM systems. AFM tapping mode was used for imaging the sample surface. It employed Si based cantilevers with Al reflective coating and a tip radius of 10 nm. Image scans were performed at each stage of the process depicted in Fig. 1. Furthermore, the scans were taken several days after the indentation where there was no pre-surface cleaning (no wet processing) performed prior to imaging. The air exposure creates surface formation of III-V oxides. We avoided HF or HCL cleaning after indentation as it can change the sample surface and affect the indent shape. Particularly, in our case we have shallow indentation in the surface.

Confocal Raman spectroscopy enables direct measurements of local mechanical stress and strain distribution in the sample. In this technique, due to its high sensitivity to modifications in the equilibrium distance between atoms, any extra phases or peak shifts can be detected. The peak positions of the Raman signal were determined by Gauss–Lorentz fitting of the measured spectra after background subtraction. The biaxial strain values were then calculated from the Raman shifts using the previously measured phonon deformation potentials for Ge. Additionally, Raman mapping of the indented area (20 µm × 20 µm) was scanned and peak filters were applied to the acquired images. The Raman 488 nm incident laser beam was precisely subjected to the indents using 100x objective lens with spot size ~0.5 μm. A low laser intensity was chosen to avoid damage to the sample.

#### Photoluminescence (PL) Measurements

The light-emission properties of the Ge islands were investigated using room-temperature photoluminescence (PL) method. This technique is well suited to study radiative recombination mechanisms of excess carriers in the Ge islands. Furthermore, the spectra hold information on the occurrence of radiative transitions and the presence of biaxial strain in the islands. The later shifts its indirect band gap into a direct one. The PL spectra of the samples were measured under 532 nm laser excitation. The laser beam was focused on the samples using 100x objective with a spot size of 0.5 µm. The emitted light was dispersed through a monochromator and measured using a room-temperature extended-range InGaAs photodetector. To increase the measurement sensitivity, a 300 grating/mm was used.
